# Molecular Dynamics Study on Thermal Conductivity Properties and Dielectric Behaviors of Graphene-Based Epoxy Resin Nanocomposites

**DOI:** 10.3390/polym17010112

**Published:** 2025-01-03

**Authors:** Chong Zhang, Chaofeng Zhao, Huize Cui, Bo Wang, Chumeng Luo, Ruilu Guo, Shuo Chen, Wenwen Gu, Wenpeng Li

**Affiliations:** China Electric Power Research Institute Co., Ltd., Beijing 100192, China; 18611602136@163.com (C.Z.); cfzhao2020@163.com (C.Z.); 18810793857@163.com (C.L.); grl18810663757@163.com (R.G.); chenshuo165546@163.com (S.C.); gww0813@outlook.com (W.G.); lwp1017@126.com (W.L.)

**Keywords:** epoxy resin, thermodynamic property, dielectric behavior, molecular dynamics simulation

## Abstract

In order to increase the thermal conductivity of neat epoxy resin and broaden its practical application in high-voltage insulation systems, we have constructed four kinds of epoxy resin nanocomposite models (a neat epoxy resin (EP), a graphene-doped epoxy resin nanocomposite (EP/GR) and hydroxyl- or carboxyl-functionalized graphene-doped epoxy resin nanocomposites (EP/GR-OH or EP/GR-COOH)) to systematically investigate their thermodynamic and electrical properties using molecular dynamics (MD) simulations. Compared with the EP model, carboxyl-functionalized graphene particles enhanced the thermal conductivity of the EP/GR-COOH model by 66.5% and increased its *T_g_* by 26.6 K. Furthermore, the dielectric constant of the EP/GR-COOH model was significantly reduced. To investigate the intrinsic mechanism, the lowest fraction of free volume (13.22%) and the largest number of hydrogen bonds (102.2) in the EP/GR-COOH model were identified as playing essential roles for its excellent thermodynamic properties and favorable electrical performance. The present study provides a molecular-level understanding of the satisfactory thermodynamic and electrical properties of the EP/GR-COOH nanocomposite, which will aid in designing novel epoxy resin nanocomposite materials with high thermal conductivity.

## 1. Introduction

Epoxy resins are widely used in the field of electrical insulation, such as basin insulators, dry-type transformers, dry-type reactors, high-voltage bushings, and insulated pull rods, because of their excellent characteristics, such as satisfactory electrical insulation properties, ease of mechanical processing, and low costs [1,2]. However, in practical applications, the low thermal conductivity of epoxy resins significantly limits their further application. In addition, a low dielectric constant is required of the materials to maintain the uniformity of the electric field and inhibit surface charge accumulation [3]. Therefore, enhancing the thermal conductivity of epoxy resins while maintaining their insulation properties can effectively improve the heat dissipation capability of electrical equipment, which is crucial for their long-term safe and stable operation [4,5].

Given the difficulty and high cost of enhancing the intrinsic thermal conductivity of epoxy resins, the most common approach is to add high-thermal-conductivity fillers to form thermal pathways within the epoxy matrix, thereby increasing the thermal conductivity of epoxy resin nanocomposites [6,7]. There are some commonly used fillers, including silica (SiO_2_) [8,9], alumina (Al_2_O_3_) [10,11], boron nitride (BN) [12,13], and graphene. Compared to other fillers, graphene exhibits exceptionally high intrinsic thermal conductivity (2000~5300 W/(m·K)) at room temperature [14]. Therefore, graphene particles can be used as an ideal filler to synthesize epoxy resin nanocomposites with high thermal conductivity. In addition, some active functional groups can be easily introduced into the graphene particle. With the introduction of hydroxyl and carboxyl functional groups, the bonding between the filler and the epoxy resin matrix can be obviously enhanced, resulting in reduced interfacial thermal resistance and increased thermal conductivity for epoxy resin nanocomposites [7]. In addition, the introduction of a graphene-based filler can also effectively improve the mechanical properties of composite materials [15].

Traditional doping and modification experiments for epoxy resin often rely on a “trial-and-error” approach [16]. It is still challenging to theoretically analyze macroscopic experimental phenomena from a microscopic perspective, and to precisely control the doping ratio of fillers. Epoxy resin performance depends on the molecular structure of the cross-linked network. Molecular dynamics (MD) simulation plays an important role in establishing the relationship between the composition, molecular structure, and properties of materials at the molecular level, as well as in the rational design and optimization of the epoxy resin. In addition, the MD simulation can also be used to address the high cost, time consumption, and numerous variable factors of traditional synthesis experiments. Recently, MD simulation has been rapidly developed and can be used to investigate the modification mechanism of novel epoxy resin nanocomposites at the molecular level [17,18]. Some researchers adopted MD simulations to theoretically study epoxy resin nanocomposites with high thermal conductivity and obtained satisfactory results [19,20]. Fu et al. [21] conducted MD simulations to investigate the relationship between the microstructure and macro-properties of diglycidyl ether of bisphenol-A (DGEBA)/methyl tetrahydrophthalic anhydride (MTHPA) and DGEBA/nadic anhydride (NA). They revealed the intrinsic mechanism of the DGEBA/NA system with a higher *T_g_* by analyzing the synergy rotational energy barrier, cohesive energy density, and fraction free volume. Wang et al. [22] adopted the DGEBA as the epoxy monomer and 1,3-benzenediamine (MPD) as the curing agent to construct a cross-linked epoxy structure. They studied changes in the thermal conductivity of nanocomposites doped with graphene and its functionalized materials using non-equilibrium molecular dynamics simulations. Their calculation results showed that the introduced amino functional groups at the edges of graphene increased the thermal conductivity of the composites by 45.3%. Ding et al. [23] constructed a cross-linked bisphenol A epoxy resin model using DGEBA and 3,3′-diaminodiphenyl sulfone (33DDS). Based on the MD simulation, they measured the theoretical thermal conductivity of pure epoxy resin (0.193 W/(m·K)), which was very close to the experimentally measured value (0.19 W/(m·K)) [24,25]. Subsequently, they used carboxyl-functionalized carbon nanotubes as fillers to construct an epoxy resin nanocomposite. The theoretical calculation results showed that the thermal conductivity of the composite system increased by 39.9% after doping with fillers. Further analysis revealed that covalent functional groups within the composites enhance the interaction between doped particles and the epoxy matrix, effectively reducing interfacial thermal resistance and thus increasing the thermal conductivity of the composite system.

**Figure 1:**
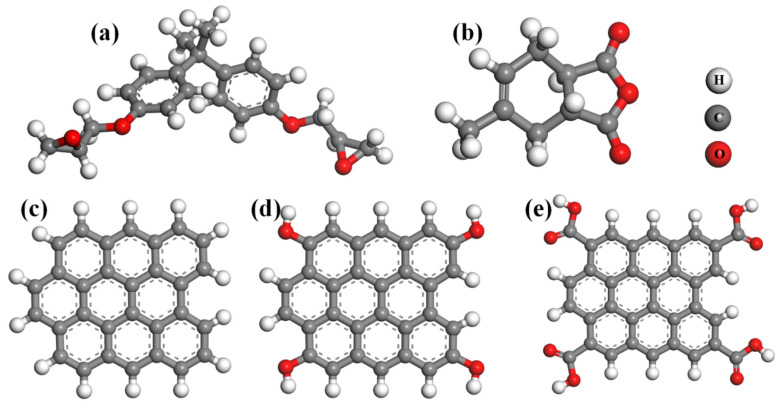
The structures of (**a**) DGEBA, (**b**) MTHPA, (**c**) a graphene particle, (**d**) a hydroxyl-functionalized graphene particle, and (**d**) a carboxyl-functionalized graphene particle.

Inspired by the aforementioned studies, this paper explores a novel epoxy resin nanocomposite that can effectively enhance thermal conductivity while maintaining good insulation properties. The MD simulations were adopted to investigate the thermodynamic properties of epoxy resin nanocomposites with graphene and its functionalized materials as fillers. In the present work, the selected epoxy monomer and curing agent were DGEBA and MTHPA, respectively, and their structures are displayed in Figure 1. Four kinds of epoxy resin nanocomposite systems were constructed, including the neat epoxy resin model (EP), a graphene-doped epoxy resin nanocomposite system (EP/GR), a hydroxyl-functionalized graphene-doped epoxy resin nanocomposite system (EP/GR-OH), and a carboxyl-functionalized graphene-doped epoxy resin nanocomposite system (EP/GR-COOH). In detail, their thermal conductivity, glass transition temperature, and dielectric constant were systematically calculated using MD simulations. We further revealed the intrinsic mechanisms of thermodynamic performance changes after filler doping through calculations of free volume fraction and hydrogen bonds. Our theoretical results can provide a theoretical basis for the synthesis of epoxy resin nanocomposites with high thermal conductivity.

## 2. Construction of Epoxy Resin Cross-Linked Model and MD Simulation Details

### 2.1. Curing Mechanism and the Design of a Cross-Linking Program

**Figure 2:**
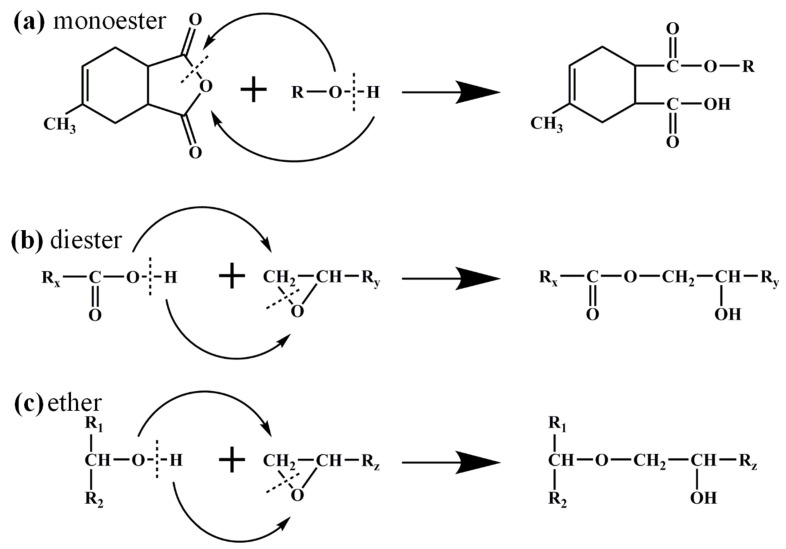
The three-step reaction mechanism of anhydride curing epoxy.

**Figure 3:**
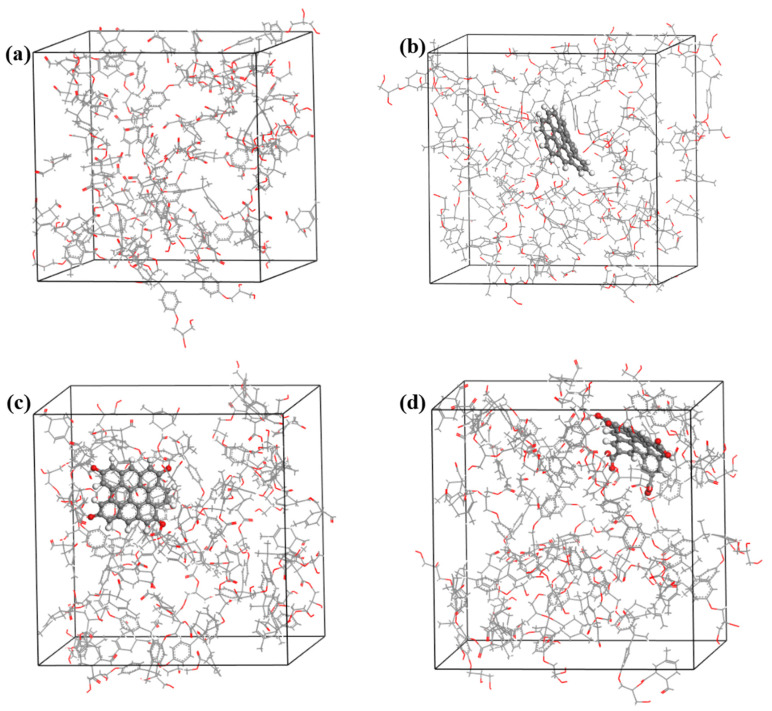
The (**a**) neat epoxy resin model (EP), (**b**) graphene-doped epoxy resin nanocomposite system (EP/GR), (**c**) hydroxyl-functionalized graphene-doped epoxy resin nanocomposite system (EP/GR-OH), and (**d**) carboxyl-functionalized graphene-doped epoxy resin nanocomposite system (EP/GR-COOH).

The accurate construction of polymer molecular models is fundamental to molecular simulation studies. The curing reaction mechanism of an anhydride curing agent with an epoxy resin monomer is shown in Figure 2 [21,26,27]. In the absence of accelerators, the reaction process between the epoxy resin monomer and the anhydride curing agent mainly includes three steps: (1) the anhydride reacts with the hydroxyl functional group in the epoxy resin to open the ring and forms a monoester (as shown in Figure 2a); (2) the carboxyl group reacts with the epoxy group to form a diester, and the generated hydroxyl group can further open the anhydride ring or react with the epoxy group in the third step (as shown in Figure 2b); (3) the hydroxyl group reacts with the epoxy group to form an ether bond (as shown in Figure 2c). Specifically, the cross-linking model of the epoxy resin in the present work is built based on the “distance judgment criterion”; that is, we search for a pair of reactive atoms within the set reaction radius, carry out the molecular dynamics equilibrium after cross-linking to obtain a stable conformation, and then continuously increase the reaction radius until the relevant conditions are met. Ultimately, the epoxy resin monomer and anhydride curing agent can form a three-dimensional network structure with high cross-linking density.

According to the reaction mechanism of anhydride-cured epoxy resin, each DGEBA molecule contains two epoxy functional groups that can react with two MTHPA molecules. Therefore, the molar ratio of DGEBA: MTHPA = 1:2. For the EP system, 20 DGEBA and 40 MTHPA molecules were randomly placed in a periodic simulation box. Based on the anhydride curing reaction mechanism, an automatic cross-linking reaction program was written in Perl, resulting in a pure epoxy resin cross-linked model with a three-dimensional network structure for subsequent molecular dynamics simulations. For the EP/GR system, since there are no additional functional groups, 20 DGEBA and 40 MTHPA molecules were also placed in the system together with one graphene fragment to construct the cross-linked structure. The percentage of the molecular weight of graphene fragment in the EP/GR model was 2.7%. As for the EP/GR-OH and EP/GR-COOH systems, since the hydroxyl- and carboxyl-functional groups in the functionalized graphene can also participate in the cross-linking reaction, 20 DGEBA and 42 MTHPA molecules were placed in the system together with functionalized graphene fragments to construct the cross-linked structure. The percentages of the molecular weight for the GR-OH and GR-COOH fragments in the EP/GR-OH and EP/GR-COOH systems were 3.1% and 3.8%, respectively. Ultimately, the structures of the four kinds of epoxy resin nanocomposites were obtained as shown in Figure 3.

### 2.2. Computational Details

After obtaining four kinds of cross-linked network models, MD simulations were performed by using GROMACS software (version 2024.3) [28,29]. Throughout the simulation process, the force field parameters about the four kinds of epoxy resin nanocomposite models were obtained from the General Amber Force Field (GAFF) [30]. Long-range electrostatic interactions were calculated using the PME method [31], while the Coulomb and van der Waals interactions were calculated using the cutoff method with a cutoff of 1.0 nm. The temperature and pressure of each system was controlled using the V-rescale thermostat [32] and Berendsen barostat [33], respectively. The time-step was 1 fs and the simulation trajectories were collected every 1 ps for analysis. For each model, the energy minimization and annealing simulation were carried out to achieve full relaxation and result in the lowest energetically stable conformation. Subsequently, MD simulations were conducted under the NPT ensemble to collect data for further analysis and calculation. The energy minimization process used the steepest descent algorithm. The annealing simulation temperature range of 600 K~300 K, and the cooling gradient of 20 K/300 ps, were adopted in the present work. The annealing simulation was cycled three times to eliminate local unreasonable structures in the models, making the system more stable and closer to the actual materials. Subsequently, reasonable conformations were obtained and can be used for subsequent simulations.

## 3. Results and Discussion

### 3.1. Relaxations of the Four Kinds of Epoxy Resin Nanocomposite Models

**Figure 4:**
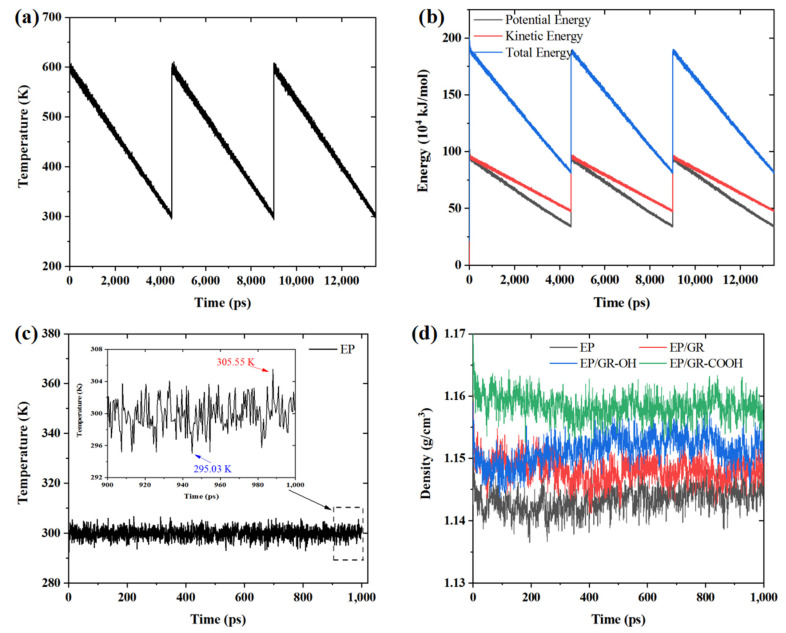
The curves for (**a**) temperature and (**b**) energy for the EP model during the annealing process. The (**c**) temperature curve of the EP system and (**d**) the density curves of the four kinds of epoxy resin nanocomposite models at relaxation at 1000 ps.

During the cyclic annealing simulation process, the energy of the system changed with the annealing temperature. Taking the EP system as an example, the temperature and energy were calculated and are shown in Figure 4a,b. From Figure 4a, we can clearly see that the temperature gradually decreased from 600 K to 300 K over three cycles of annealing. In Figure 4b, the potential energy, kinetic energy, and total energy of the EP system also exhibited periodic changes during the annealing simulation process.

There are two typical criteria for determining whether a system has reached equilibrium: (1) the temperature of a system reaches a stable state, and the temperature fluctuations within 15 K after relaxation; or (2) the energy of a system reaches a stable state, indicated by the energy values fluctuating around a fixed value. After cyclic annealing treatment for the four kinds of epoxy resin nanocomposite systems, these models were subsequently relaxed for 1000 ps under the NPT ensemble. The temperature fluctuation curve of the EP system was shown in Figure 4c. From Figure 4c, the temperature of the EP system maintained stabilization during the MD simulation progresses. By selecting the last 100 ps of the MD simulated trajectory, the maximum temperature fluctuation amplitude of 10.52 K could be observed clearly, meeting the convergence conditions for system equilibrium. In addition, we also calculated the total energy of each system. The calculated energy fluctuation range of the EP system was stable within 2.99% and the energy fluctuation range of the other three epoxy resin nanocomposite systems was also stable within 3.53%. All of these systems reached the convergence conditions of energy equilibrium. Hence, we calculated the density curve of each system and summarize them in Figure 4d. After the systems reached equilibrium through the dynamic relaxation process, the densities of the EP, EP/GR, EP/GR-OH, and EP/GR-COOH systems were 1.144 g/cm³, 1.148 g/cm³, 1.152 g/cm³, and 1.158 g/cm³, respectively. In particular, the density of the EP system was consistent with the reported density values in the literature [21], which verified that the constructed model in present work was reliable.

### 3.2. The Calculation of Thermal Conductivity

Heat conduction is the thermodynamic phenomenon of energy transfer caused by a temperature gradient. The heat conduction process is a non-equilibrium. In the present work, the non-equilibrium molecular dynamics method, based on Fourier’s law, was adopted to calculate the thermal conductivity of the four kinds of epoxy resin nanocomposite models. At first, a temperature gradient was established. Once the system reached a steady state, the temperature gradient and non-equilibrium steady state heat flux of the system could be obtained. Then, the thermal conductivity could be calculated according to Fourier’s law. The thermal conductivity calculation formula based on Fourier’s heat transfer is shown in Equations (1)–(3):

(1) $$\kappa =-\frac{J}{A\nabla T}$$

(2) $$J=\frac{\partial M}{\partial t}$$

(3) $$\nabla T=\frac{\partial T}{\partial x}$$

where *J* is the non-equilibrium heat flux, *A* is the heat transfer area, *T* is the temperature, ∇T is the temperature gradient, *M* is the average absolute value of energy inflow from heat sources and sinks, t is the simulation time, and x is the direction of heat conduction.

In this part, the thermal conductivities in the x, y, and z directions for the EP, EP/GR, EP/GR-OH, and EP/GR-COOH models were calculated, respectively. The arithmetic mean of the thermal conductivities in three directions was taken as the overall thermal conductivity of the structure. The results are summarized in Table 1. From Table 1, it can be seen that the overall thermal conductivity of the EP system was 0.176 W/(m·K), which was close to the experimentally reported 0.19 W/(m·K) [24,25]. The overall thermal conductivity of EP/GR was 0.173 W/(m·K), showing no significant change compared to the EP system. In comparison, the overall thermal conductivities of the EP/GR-OH (0.205 W/(m·K)) and EP/GR-COOH (0.293 W/(m·K)) systems significantly increased, which improved by 16.7% and 66.5%, respectively. The hydroxyl and carboxyl functional groups of the introduced graphene fillers could form hydrogen bonds with the epoxy resin matrix to enhance the interaction between the doped particles and the epoxy resin matrix. Finally, the interfacial thermal resistance could be effectively reduced, resulting in significant improvement in the thermal conductivity of the composite system.

### 3.3. The Calculation of Glass Transition Temperature

**Figure 5:**
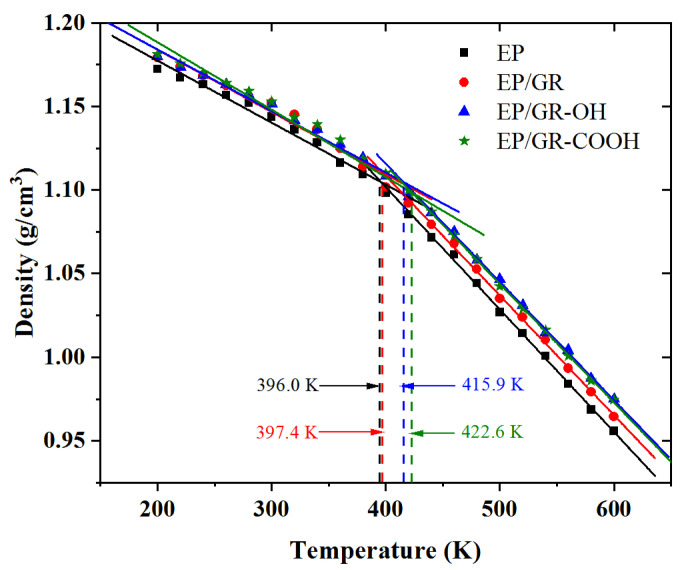
The density of each epoxy resin nanocomposite model as a function of temperature.

The glass transition refers to the change in amorphous substances between the glassy state and the rubbery state. The glass transition temperature (*T_g_*) is typically used to characterize the ability of a polymer to withstand external temperatures. Thereby, the *T_g_* can be used to reflect the thermal stability of the polymer. Since the density of a material changes significantly before and after the glass transition temperature, the “density–temperature curve” method can be used to obtain the *T_g_* of the material. In the present work, a simulated quasi-static cooling process was carried out to establish the density–temperature curve of each epoxy resin nanocomposite model. Firstly, the system temperature was set at 600 K, and a 1000 ps dynamic relaxation process was conducted under the NPT ensemble to eliminate internal imbalances and minimize the system energy. Then, in the temperature range of 600~200 K with intervals of 20 K, a 500 ps dynamic simulation was performed at each temperature under the NPT ensemble. The density data of the system were extracted from the trajectory to plot the density scatter plot of the system at different temperatures. Subsequently, a linear fitting method was used to obtain two fitted lines for the low-temperature and high-temperature segments. As shown in Figure 5, the x-coordinate of the intersection of these two fitted lines was *T_g_*. From Figure 5, it can be seen that the *T_g_* of the EP system was 396.0 K, which was consistent with the experimentally reported *T_g_* value in the range of 390~402 K [21,34]. The *T_g_* of the EP/GR system was 397.4 K, which was slightly higher than that of the EP system. As for the EP/GR-OH and EP/GR-COOH systems, their *T_g_* were 415.9 K and 422.6 K, respectively, showing significant increases compared to the EP system (396.0 K). The main reason was that the incorporation of fillers in the epoxy resin system compressed its original space, thereby slowing down the movement tendency of the epoxy resin and improving the thermal stability of the system. In contrast, the EP/GR-COOH system exhibited the highest *T_g_* because the carboxyl functional groups on the graphene could form hydrogen bonds with the epoxy resin matrix to enhance the intermolecular interactions and significantly improve the stability of the system.

### 3.4. The Calculation of Structural Parameters

To further investigate the intrinsic mechanism of the enhanced thermodynamic performance for the epoxy resin nanocomposites doped with graphene-based fillers, the fraction of free volume (*FFV*) and hydrogen bonds of the four kinds of systems were further calculated.

The internal total volume of polymer materials consists of the occupied volume and the free volume. The occupied volume refers to the space occupied by molecular chains within the material, while the free volume refers to the space where molecular chains can move freely. Therefore, the fraction of free volume (*FFV*) of a polymer has a certain impact on its thermodynamic performance. The *FFV* can be calculated based on the following formula:

(4) $$FFV=\frac{V_{f}}{V_{0}+V_{f}}\times 100\%$$

where $V_{f}$ represents the free volume of the system and $V_{0}$ represents the occupied volume of the system.

**Figure 6:**
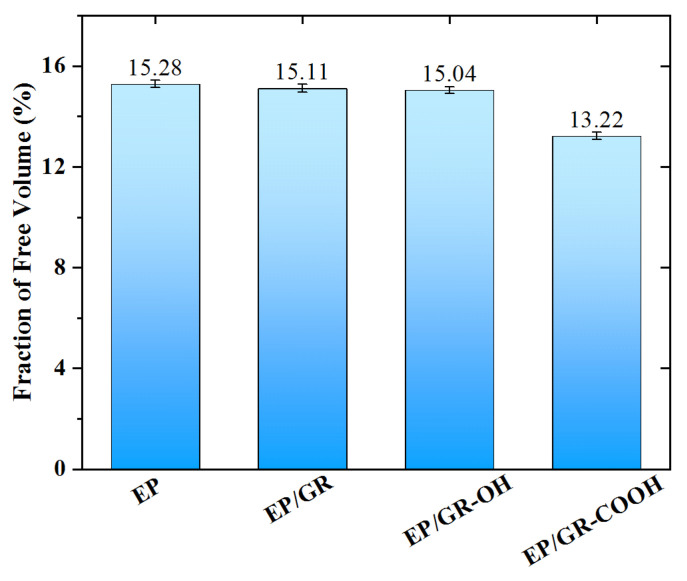
The fraction of free volume in the four kinds of epoxy resin nanocomposite models.

Before cross-linking, the small molecular chains of DGEBA and MTHPA have relatively strong motility and can move freely in the network. Therefore, the free volume of the system is large. When the cross-linking starts, the number of monomer molecules will decrease and result in a significant decrease in *FFV*. In this section, the *FFV* of the four kinds of epoxy resin nanocomposite models in this study was calculated, and the results are shown in Figure 6. From Figure 6, it can be observed that compared to the EP system (15.28%), the *FFV* of the systems with doped fillers gradually decreased. Especially, the EP/GR-COOH system exhibited the lowest *FFV* value (13.22%). This result indicated that the doped graphene-based nanoparticles could fill the voids of the epoxy resin matrix and reduce the mobility of molecular chains in the epoxy resin matrix. The lower *FFV* of a structure was beneficial to maintain its structural stability at the high temperature, resulting in a high *T_g_* [35].

Additionally, the hydrogen bond network in the polymer material is closely related to its macroscopic parameters, such as its melting point, boiling point, and molecular conformation. And the hydrogen bond network is an important factor in determining the mechanical strength and thermal stability of the polymer. Therefore, the number and lifetime of hydrogen bonds in the four epoxy resin nanocomposite models were calculated, and the results are summarized in Table 2. Compared with the EP system (99.9), the number of hydrogen bonds in the EP/GR (79.4) and EP/GR-OH (74.6) systems slightly decreased. In comparison, the hydrogen bonds in the EP/GR-COOH system exhibited the largest number and the longest lifetime, which indicated that hydrogen bonding was a major factor affecting the thermodynamic stability of the epoxy resin nanocomposite models.

### 3.5. The Calculation of Dielectric Constant

The application of epoxy resin in the field of electrical insulation requires not only high thermodynamic stability, but also favorable insulation requirements. The dielectric constant can be derived by calculating the dipole moment, as shown in the following equation:

(5) $$\varepsilon_{r}=1+\frac{1}{3Vk_{B}T\varepsilon_{0}}\left(\langle M^{2}\rangle -{\langle M\rangle }^{2}\right)$$

where *M* is the dipole moment of the system at a time step, $\langle M^{2}\rangle$ is the average for the sum of the squares for each dipole moment in the molecular system trajectory, ${\langle M\rangle }^{2}$ is the square of the average of all dipole moments in the trajectory, *V* is the volume, *k_B_* is the Boltzmann constant, and $\varepsilon_{0}$ is the vacuum permittivity.

To explore the effect of doped graphene-based nanoparticles on the insulation performance of epoxy resin, the dielectric constant of the four kinds of epoxy resin nanocomposite models in this study was calculated according to Equation (5), as shown above. As shown in Table 3, the dielectric constant of the EP system was 4.96, and it decreased to 4.53 after doping with graphene in EP/GR system. This phenomenon could be attribute to the strong charge transfer and adsorption capabilities of graphene particles, resulting in reduced dipole moment fluctuations for the epoxy resin matrix. However, after doping with hydroxyl-functionalized graphene, the dielectric constant of the EP/GR-OH system significantly increases to 5.80. The introduced polar hydroxyl functional groups could enhance the dipole moment of the system, thereby increasing the overall dielectric constant. Although carboxyl was also a polar functional group, the dielectric constant of the EP/GR-COOH system (3.11) was much lower than that of the EP/GR-OH system (5.80). Combined with the aforementioned analysis about hydrogen bonds, the number and strength of formed hydrogen bonds in the EP/GR-COOH system were higher than that of the EP/GR-OH system. Therefore, the hydrogen bonds constrained the rotational motion and reduced the dipole moment fluctuations of the epoxy resin matrix, resulting in a decreased dielectric constant [36]. In summary, it can be found that epoxy resin nanocomposite doped with carboxyl-functionalized graphene particles can still maintain excellent insulation performance.

## 4. Conclusions

The thermodynamic and electrical properties of the four kinds of cross-linked epoxy resin/functionalized graphene nanocomposite models were systematically investigated using the MD simulations. The following conclusions were drawn from this study.

(1) Compared with the neat EP system, the overall thermal conductivity and *T_g_* of EP/GR-COOH system increased by 66.5% and 26.6 K, respectively.
(2) The dielectric constant of the EP/GR-COOH system is obviously lower than that of the EP system, providing excellent insulation protection.
(3) The large number of hydrogen bonds and lower fraction of free volume for the EP/GR-COOH system play important roles in its excellent thermodynamic properties and satisfactory electrical performance.

In summary, EP/GR-COOH, with high thermal conductivity and a low dielectric constant, can be a promising material for the application of high-voltage insulation systems. Our fundamental study can provide a theoretical basis for the doping design and performance regulation of novel epoxy resin composites.

## Figures and Tables

**Table 1:** The thermal conductivity of the epoxy resin nanocomposite models.

System	Thermal Conductivity/[W/(m·K)]			
	x Direction	y Direction	z Direction	Overall
EP	0.187	0.172	0.169	0.176
EP/GR	0.183	0.177	0.159	0.173
EP/GR-OH	0.195	0.225	0.197	0.205
EP/GR-COOH	0.336	0.291	0.252	0.293

**Table 2:** The number and lifetime of hydrogen bonds in each epoxy resin nanocomposite model.

System	Number	Lifetime/ps
EP	99.9	2.99
EP/GR	79.4	3.00
EP/GR-OH	74.6	3.05
EP/GR-COOH	102.2	3.40

**Table 3:** The dielectric constant of each epoxy resin nanocomposite model.

System	Dielectric Constant
EP	4.96
EP/GR	4.53
EP/GR-OH	5.80
EP/GR-COOH	3.11

## Data Availability

The original contributions presented in this study are included in the article. Further inquiries can be directed to the corresponding author.
